# Organoids in domestic animals: with which stem cells?

**DOI:** 10.1186/s13567-021-00911-3

**Published:** 2021-03-04

**Authors:** Bertrand Pain

**Affiliations:** Univ Lyon, Université Lyon 1, INSERM, INRAE, Stem Cell and Brain Research Institute, U1208, CSC USC1361, Bron, France

**Keywords:** Pluripotent stem cells, Adult stem cells, Induced pluripotent stem cells, Organoids

## Abstract

Organoids are three-dimensional structures that are derived from the self-organization of stem cells as they differentiate in vitro. The plasticity of stem cells is one of the major criteria for generating organoids most similar to the tissue structures they intend to mimic. Stem cells are cells with unique properties of self-renewal and differentiation. Depending on their origin, a distinction is made between pluripotent (embryonic) stem cells (PSCs), adult (or tissue) stem cells (ASCs), and those obtained by somatic reprogramming, so-called induced pluripotent stem cells (iPSCs). While most data since the 1980s have been acquired in the mouse model, and then from the late 1990s in humans, the process of somatic reprogammation has revolutionized the field of stem cell research. For domestic animals, numerous attempts have been made to obtain PSCs and iPSCs, an approach that makes it possible to omit the use of embryos to derive the cells. Even if the plasticity of the cells obtained is not always optimal, the recent progress in obtaining reprogrammed cells is encouraging. Along with PSCs and iPSCs, many organoid derivations in animal species are currently obtained from ASCs. In this study, we present state-of-the-art stem cell research according to their origins in the various animal models developed.

## Introduction

**Figure 1:**
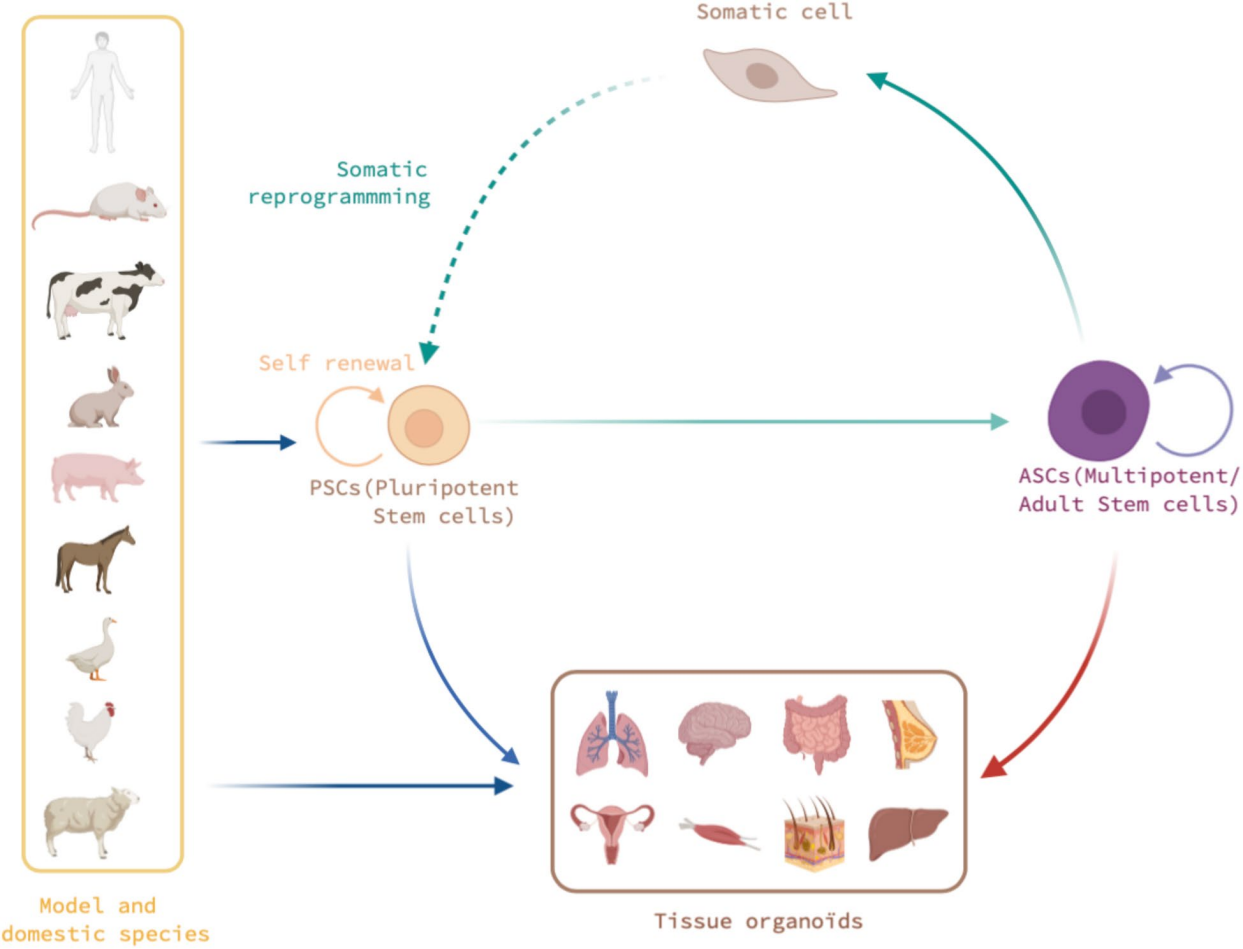
**Schematic illustration of the various stem cells that could be used to generate 3D organoids, depending on their origin and species**. Pluripotent stem cells (PSCs) exhibit unique self-renewal and differentiation properties. Derived from embryos or obtained through somatic reprogramming, PSCs have been obtained in model species (human, rodents) and in some domestic birds and mammals. Currently, the PSCs isolated from these mammalian species do not exhibit the same cell plasticity or differentiation properties as those of model and avian species. Multipotent stem cells are usually assimilated to the adult stem cells (ASCs) found in embryonic and adult tissues, such as hematopoietic, intestinal, neural, or dermal stem cells. These cells are presently the major sources for deriving organoids in domestic animals. Among the organoids of interest, we will illustrate in the next chapters of this review those developed for the brain, intestines, liver, lungs, mammary glands, muscles, reproductive system, and skin.

In part I, we present a summary of the state-of-the-art models of pluripotent stem cells (PSCs), adult stem cells (ASCs), and stem cells induced in two original murine and human models with the data available in the mammalian agricultural domestic species of interest, and also in birds. A schematic figure illustrates the interactions between stem cells, organoids, and species (Figure 1). In part II, we summarize the main fields of application of organoids, the use of which will be described in more detail in the following chapters, which are dedicated to several tissues of interest.

## Part I: organoids and 3D structures established in mouse and human models

### Definition

An organoid is defined as a three-dimensional structure that self-organizes from a PSC or an ASC and has the capacity to self-renew and to differentiate to give rise to the different constitutive morphotypes of the tissue it aims to mimic and to reproduce at least some of its physiological functions. This functional notion becomes preponderant in the definition of an organoid derived from stem cells and differentiates organoids from explants, which maintain the preexisting functions of an already developed tissue. This same notion of functionality of organoids also differentiates them from embryoid bodies, obtained by the aggregation of PSC cells but according to a mode of non-directed differentiation and which do not self-organize. Different types of stem cells can generate organoids, depending on their developmental or tissue origin [1]. In a very general description, the main steps to generate an organoid are the initial proliferation of stem and precursor cells in a 3D structure and then their induction in an environment which allows it to differentiate under the effect of inducers (media, growth factors, chemical molecules, etc. …). These changes in culture conditions are either put together or sequentially to guide stem cell differentiation. The whole process ensures the development and maturation of the organoid which then presents several cellular morphtypes representative of the tissue. Cancer stem cells can also help produce 3D tumors in certain conditions, but are not considered in the context presented here.

### Embryonic PSCs

One of the goals of cell culture is to mimic the functions of the tissue from which the cells are taken. Whether it is healthy or pathological tissue, the objective is to have an in vitro model to study the physiological functions or the pathological state of the tissue. Numerous cell lines have been isolated and characterized, including stem cells derived either from embryos to obtain embryonic stem cells (ESCs) or from tissues to obtain ASCs. ESCs were first isolated in the 1980s in the mouse model [2, 3]. Characterized by their self-renewal and their potential for differentiation in vitro, mouse ESCs (mESCs) also have the unique property of contributing to the somatic and germinal chimerism of an embryo when they are injected into a preimplantation blastocyst. Non-human and human primate ESCs were first obtained in the 1990s [4, 5] from the culture of preimplantation embryos. The culture conditions remain the critical point in the establishment of these cells and their plasticity, depending on the species. In the 2000s, epiblast stem cells (EpiSCs) were isolated from a postimplantation mouse embryo [6, 7]. These cells no longer have the property of contributing to chimerism in vivo even if they retain the properties of differentiating in vitro in the three embryonic lineages (the ectoderm, the endoderm, and the mesoderm). This property of colonization of the embryo and, in particular, of germinal colonization is currently considered as one of the most stringent criteria, making it possible to distinguish ESCs in a state known as “naive” versus the “primed” state, whose archetype are EpiSCs [8]. Numerous publications have characterized these two types of stem cells, which also differ in their culture conditions and in their molecular and epigenetic characterization [9–11].

The existence of these same stages in species other than rodents is still widely debated, especially in human and non-human primate models, for which many works have tried to define culture conditions to obtain and maintain naive cells [12]. In particular, the use of different cocktails of small molecules that inhibit signaling pathways have been described, which make it possible to obtain naive cells [13–15].

In other species, ESCs have been isolated, but this strict definition of germ colonization is currently restricted to rodents. We can therefore undoubtedly qualify these cells as embryonic stem (ES)-like cells in the absence of this fundamental property, in particular for mammalian species other than rodents. These “ES-like” cells have been isolated, amplified, and established in lines with self-renewal and differentiation properties in many species, including agricultural species such as pigs [16–19], cows [20, 21], sheep and goats [22–24], horses [25, 26] and rabbits [27, 28]. PSCs have been obtained and validated in birds, such as chickens [29, 30], and in fish such as medaka [31, 32] and zebrafish [33]. Most of these cells were characterized by their proliferation and differentiation potential in vitro and by the presence of certain markers, such as surface antigens, including SSEA1, SSEA3, and SSEA4, antigens initially identified in mice but whose cross-reactivity with other species has been found to be important in identifying these cells [34, 35]. Among these cells, very few have the property of colonization of the embryo, even at the somatic level, with the notable exceptions of chicken [29, 30] and zebrafish cells [33].

More recently, using strategies similar to those developed for human cells based on cocktails of inhibitory molecules, studies have been conducted of obtaining ESCs in bovine and porcine species even if chimera experiments were not reported [18, 20, 21]. In addition, recent molecular analyses carried out at the level of cells isolated from preimplantation embryos have made it possible to better define the markers associated with these early stages in species such as cattle, pigs, and rabbits and to compare them with mouse, primate, and human models [36–38]. These studies should help the development of cells with properties close to or identical in their developmental properties to those of murine naive cells.

### Adult stem cells

ASCs are multipotent cells that can generate all the specialized cell types that are present in the specific tissue or organ from which they have been isolated. They have been isolated progressively from most human tissues because of the characterization of numerous markers specific to each tissue niche. Hematopoietic stem cells were the first to be identified, purified, and characterized in the 1960s by the pioneering work of Till and McCullough [39]. Mesenchymal stem cells were also very quickly identified in the same bone marrow tissue, and their presence in a multitude of tissues was confirmed subsequently, even if the nature of the markers to identify them varies. Neural stem cells were also identified in the 1960s from fetal brain tissue and later, in the 1990s, in adult brain tissue. The isolation of neurospheres under well-defined culture media conditions allowed their culture [40]. Progressively, stem cells from other tissues have been isolated and characterized, notably with the pioneering work of H. Clevers on the intestinal and epithelial stem cells of numerous organs and the identification of *LGR5* as a marker for many of these cells [41]. To date, stem cells have been identified in most organs [42, 43], including the skin [44], muscles [45], the intestine [46], the liver [47], the lungs [48, 49], the mammary glands [50], and the reproductive system, for which the reproductive cells also have very particular properties of self-renewal and differentiation. Organoids derived from ASCs are generated without genetic modification by transcription factors, unlike organoids derived from iPSCs.

### Induced PSCs

The arrival of somatic reprogramming technology in 2006 truly revolutionized the field of stem cell research [51–53]. Induced PSCs (iPSCs) share properties similar to ESCs without the inconvenience of the ethical issues (at least for the human species). Initially demonstrated in the mouse model, the concept was extended quickly to non-human primates, humans [54], and many other species, including rabbits [55, 56], sheep and cattle [57–60], pigs [18, 61–63], dogs [64, 65], cats [66], horses [67, 68], and even some endangered species such as the snow leopard [69]. However, in these animal species, it is difficult to validate the status of the reprogrammed cells [70] because the developmental potential of the reprogrammed cells is rarely tested. In non-mammalian species, the results are scarcer for avian cells [71–73] and tests on other species [72]. Nevertheless, the main interest in iPSCs, in particular for humans but also for agricultural species, lies in obtaining cells with properties similar to ESCs, but without going through the derivation from embryos. Even if the conditions of derivation and maintenance by different culture media are not yet conducive to obtaining the most plastic cells possible in agricultural species, progress is being made constantly, providing hope to obtaining such cells in a timely and reasonable manner.

The concept of somatic reprogramming is to introduce a cocktail of genes –including transcription factors involved in the control of pluripotency–into a somatic cell, thus leading to its reprogramming to a pluripotent cell. The first canonical combination (*OSKM, OCT4, SOX2, KLF4,* and *c-MYC* genes) was described by S. Yamanaka’s team in 2006. Subsequently, other combinations and genes have been identified gradually, such as the *OCT4, SOX2, NANOG,* and *LIN28 (OSNL*) combination [54] and actors such as Nr5a2 [74], *ESRRB* [75], *GLIS1* [76], *ZIC3* [77], *TBX3* [78], *H1f00* [79], *NKX3.2* [80], and miR302 [81]. These factors either participate directly in the reprogramming process or increase the reprogramming efficiency [82, 83]. Surprisingly, it was recently reported that the absence of OCT4 may favor reprogramming [84]. The mechanisms controlling the epigenetic barriers that exist between somatic and pluripotent cells have also been taken into account to better understand and facilitate reprogramming [85–87]. Many methods and molecules have been described to have a positive or negative impact in the reprogramming stages [88–90].

### Initial establishment of organoids

The self-renewing and differentiating properties of ESCs or iPSCs as well as ASCs are the two critical properties for the generation of organoids. The pioneering work of the laboratory of Y. Sasai [91] and then of H. Clevers [41] was quickly popularized by the development of numerous approaches for the production of organoids in most tissues. Initially developed from tissue stem cells, more complex protocols also appeared from PSCs. Most of these studies were carried out in mouse and human models and, so far, little data are available for other species, especially those of agricultural interest [92].

In this review, we focus on the achievements and projects carried out in non-human and rodent species, with special focus on species of agricultural interest.

## Part II: biological issues addressed by 3D organoids

In recent years, the significant development of 3D organoid approaches has allowed various applications. Human organoids were first presented as tools to study tissue development with pioneering achievements (retina, intestine, etc.) [93]. Soon after, they were used as models to mimic and reproduce certain pathologies in vitro [43, 94]. In particular, the development of brain organoids has paved the way for the modeling of many neurodegenerative pathologies, for which access to brain tissue is almost impossible. The possibility of deriving reprogrammed human-induced PSCs (hiPSCs) from patient biopsies, modifying them, and obtaining isogenic cells by reversing mutations through genome editing approaches, for example, has also allowed numerous studies and publications for a large spectrum of pathologies that affect the nervous system [94–98]. The same hiPSCs from patients with genetic diseases have been used to generate brain organoids and to model the impact of the mutation that is observed in the patient during the development of these structures with examples such as the RETT syndrome or amyotrophic lateral sclerosis [97–99].

If the analysis of mutations is a privileged axis at both the developmental and pathological levels for congenital genetic alterations in particular, the organoid approach also makes a contribution to obtain models for studies in oncology, in particular, through the potential development of personalized medicine to adapt treatment for individual patients with cancer. This new methodology has already been implemented in the intestine, kidney, prostate, ovary, bladder, pancreas, liver, breast, and brain [100–103] and new models and uses are likely to emerge. Through the development of tumoroids derived from patient biopsies, it may be possible to test the most efficient molecules on diseased organoids in parallel with healthy organoids obtained from the same patient and therefore to better adapt a targeted antitumor treatment and target tumoral cells in the patients. The published results are encouraging [104–106]. Unthinkable only a few years ago, the first demonstrations are underway to screen certain anticancer agents and adapt treatment to each patient.

Owing to their near-physiological state, organoids are promising innovative tools for toxicological studies. Their low ethical concerns, as compared with in vivo animal studies, also argue in favor of their use in this field. They have already been used to assess the toxicity of many substances in humans. It is probable that animal-based organoids will also be used more widely in the future to predict the adverse effects of drugs in the target species. Similarly, toxicological screening approaches are particularly concerned by this 3D organoid approach to better define the toxicological threshold of a molecule in an environment different from that of an adherent line or culture without complexity, therefore trying to fit with what is observed in a tissue. If the difficulty is to operate with a “floating” structure and one that takes longer to obtain than the simple seeding of an adherent cell line, the answers are more relevant as to the availability of the molecules tested within the tissue.

Studies of infectious diseases have been limited by the paucity of functional models that mimic normal physiology and pathophysiology in a species-specific manner. The development of brain, lung, and intestine organoids, among others, of human and animal species constitutes a considerable advance that facilitates studies of host–pathogen interactions. Breakthroughs in their understanding, for viruses as well as bacteria and parasites, are greatly expected. In the dedicated sections, examples will be given of some of the major discoveries that have been made in this field thanks to organoids. Another area that has received special attention is the development of approaches to study pathogen–host relationships, whether these pathogens are bacteria [107], parasites [108], or viruses [109, 110], by targeting different tissue models via brain organoids [109], intestinal organoids [111], or pulmonary organoids [107, 111, 112], among others. For example, approaches to the infection of cerebral organoids by the Zika virus have highlighted the tropism of this virus for neuronal precursors and therefore make an a priori link with the microcephaly observed in infants following the infection of mothers [109, 113, 114]. More recently, the same approach was put forward to follow the impact of the viral spread of largely unknown viruses, such as SARS-Cov-2. The tissue complexity reproduced at the organoid scale makes it possible to study the propagation of the pathogen in all the tissue components, in particular to reproduce the kinetics of contamination [115–117]. In an original manner, the organoids, in this case intestinal, can make it possible to compare different susceptibilities to SARS-Cov-2 between human and bat models [118]. Mention should also be made of studies on microbiota–host interactions, whether these are normal or pathogenic. The study of microbiota has become important in many developmental and pathological aspects and in several tissues, although the intestine remains the reference tissue for these approaches. The skin, the lungs, etc., also have their own microbiota and having in vitro models to test the balance and imbalance of these ecosystems is relevant.

## Conclusion

In the rapidly developing field of organoids, the human model is still the most studied, but many new developments concern agricultural animals. At this level, having physiological in vitro models that closely mimic whole tissues, but are different from explants that require repeated biopsies, will be an advantage. The development of organoids also responds to the increasingly significant and important societal demand to limit animal testing.

## Abbreviations

ASCs: Adult stem cells
EpiSCs: Epiblast stem cells
ESCs: Embryonic stem cells
iPSCs: Induced pluripotent stem cells
PSCs: Pluripotent stem cells
